# Role of Osteopontin as a Potential Biomarker of Pulmonary Arterial Hypertension in Patients with Systemic Sclerosis and Other Connective Tissue Diseases (CTDs)

**DOI:** 10.3390/ph14050394

**Published:** 2021-04-21

**Authors:** Mattia Bellan, Cristina Piccinino, Stelvio Tonello, Rosalba Minisini, Ailia Giubertoni, Daniele Sola, Roberta Pedrazzoli, Ileana Gagliardi, Erika Zecca, Elisa Calzaducca, Federica Mazzoleni, Roberto Piffero, Giuseppe Patti, Mario Pirisi, Pier Paolo Sainaghi

**Affiliations:** 1 Department of Translational Medicine, Università del Piemonte Orientale UPO, 28100 Novara, Italy; stelvio.tonello@uniupo.it (S.T.); rosalba.minisini@uniupo.it (R.M.); ailia.giubertoni@maggioreosp.novara.it (A.G.); ileanagagliardi91@gmail.com (I.G.); erikazecca24@yahoo.it (E.Z.); elisa.calzaducca91@gmail.com (E.C.); 20009581@studenti.uniupo.it (F.M.); 20009551@studenti.uniupo.it (R.P.); giuseppe.patti@med.uniupo.it (G.P.); mario.pirisi@med.uniupo.it (M.P.); pierpaolo.sainaghi@med.uniupo.it (P.P.S.); 2 Division of Cardoilogy, “AOU Maggiore della Carità” Hospital, 28100 Novara, Italy; cristina.piccinino@maggioreosp.novara.it (C.P.); daniele.sola@med.uniupo.it (D.S.); roberta.pedrazzoli@maggioreosp.novara.it (R.P.); 3 CAAD (Center for Translational Research on Autoimmune and Allergic Disease), Maggiore della Carità Hospital, 28100 Novara, Italy

**Keywords:** osteopontin, pulmonary arterial hypertension, systemic sclerosis, connective tissue diseases

## Abstract

Pulmonary arterial hypertension (PAH) is a severe complication of connective tissue diseases (CTD). Its early diagnosis is essential to start effective treatment. In the present paper, we aimed to evaluate the role of plasma osteopontin (OPN) as a candidate biomarker of PAH in a cohort of CTD patients. OPN is a pleiotropic protein involved in inflammation and fibrogenesis and, therefore, potentially promising in this specific clinical context. We performed a cross-sectional observational study on a cohort of 113 CTD patients (females N = 101, 89.4%) affected by systemic sclerosis N = 88 (77.9%), mixed connective tissue disease N = 10 (8.8%), overlap syndrome N = 10 (8.8%) or undifferentiated connective tissue disease N = 5 (4.4%). CTD-PAH patients showed significantly higher OPN plasma values than patients with CTD alone (241.0 (188.8–387.2) vs. 200.7 (133.5–281.6) ng/mL; *p* = 0.03). Although OPN levels were directly correlated with age and inversely with glomerular filtration rate, they remained associated with PAH at multivariate analysis. In conclusion, OPN was significantly associated with PAH among patients with CTD, suggesting it may have a role as a non-invasive disease biomarker of PAH.

## 1. Introduction

Pulmonary arterial hypertension (PAH) is a relatively uncommon condition, defined by the presence of a mean pulmonary arterial pressure (mPAP) equal to or greater than 25 mmHg assessed during invasive right heart catheterization (RHC) at rest; PAH is defined precapillary when pulmonary capillary wedge pressure (PCWP) equal to or less than 15 mmHg, [1]. PAH is a severe and potentially life-threatening complication of systemic sclerosis (SS) and scleroderma spectrum disorders (SSD), a definition encompassing clinical entities sharing common features with SSc: mixed connective tissue diseases (MCTD) and SS overlap with other connective tissue diseases (CTDs) [2]. The CTD-associated PAH (CTD-PAH) carries a worse prognosis than the idiopathic PAH [3].

The early diagnosis of PAH in a patient affected by a CTD is crucial but requires a high degree of suspicion since PAH is initially minimally symptomatic or asymptomatic. The two-step algorithm DETECT is the most widely used screening tool for SS patients [4]. However, novel diagnostic biomarkers and PAH predictors are needed [5,6], being a timely diagnosis quintessential to early treatment and improved prognosis [7].

Osteopontin (OPN) is a 32-kDa secreted, extracellular-matrix glycosylated phosphoprotein encoded by a gene located on chromosome 4 (4q13) with pleiotropic effects, among which regulation of the inflammatory response is paramount [8]. Indeed, systemic inflammatory disorders are associated with an increase in OPN plasma levels. For instance, patients with rheumatoid arthritis have high OPN concentrations [9]; similarly, OPN levels are increased in sepsis and have a prognostic value [10]. This increase follows the release of OPN by macrophages, activated T cells, endothelial and epithelial cells during the inflammatory response [8]: OPN then acts as a chemoattractant, assisting the recruitment of immune cells in the inflamed tissues [11].

Moreover, OPN is implicated in regulating fibrogenesis [12,13], with growing evidence linking this molecule specifically to the pathogenesis of dermal fibrosis in SSc [14]. Finally, OPN plays a role in the vascular remodeling process [15], and its levels are increased in patients affected by PAH. Importantly, OPN is one of the top five overregulated genes in explanted lungs of PAH patients, independently from what caused PAH, but with a direct correlation with the severity of the disease [16].

Inflammation, fibrosis and vascular remodeling are major pathogenetic mechanisms driving developing PAH during CTD clinical course. Therefore, OPN may represent a promising candidate biomarker of PAH among CTD patients; the present pilot study was built to verify this hypothesis.

## 2. Results

The study population included 101 females (89.4%) and 12 males (10.6%), with a median age of 65 years (54–74). These patients were classified as follows: SSc, N = 88 (77.9%); MCTD, N = 10 (8.8%); overlap syndrome, N = 10 (8.8%); UCTD, N = 5 (4.4%). Of the 88 patients with SSc, 68 (77.3%) were classified as limited cutaneous SSc and 20 (22.7%) with the diffuse variant. Table 1 presents the main clinical features and main antirheumatic ongoing treatment of the study population.

Sixteen patients (14.2%) were diagnosed with CTD-PAH (all the patients received a diagnosis of type 1 pulmonary hypertension; however, 2/16 showed mixed pathogenesis, type 1 and 3). The diagnosis was established by RHC in 15/16 patients. The mean pulmonary arterial pressure (PAP) was 30 (26–36) mmHg, with a median pulmonary vein resistance of 4.1 (3.3–6.4) WU.

As shown in Table 1, PAH patients were significantly older than those with CTD. In Table 2, we report the differences among groups concerning laboratory and instrumental findings.

Looking at the laboratory parameters, patients with CTD-PAH showed lower Hb and eGFR; conversely, BNP was significantly higher. As expected, CTD-PAH patients have a reduced DLCO, while other pulmonary function tests were not statistically different from those observed in the other CTD patients. Moreover, CTD-PAH patients had higher PAPS.

Disease duration, years: 10.5 (4.0–14.0)

**Figure 1:**
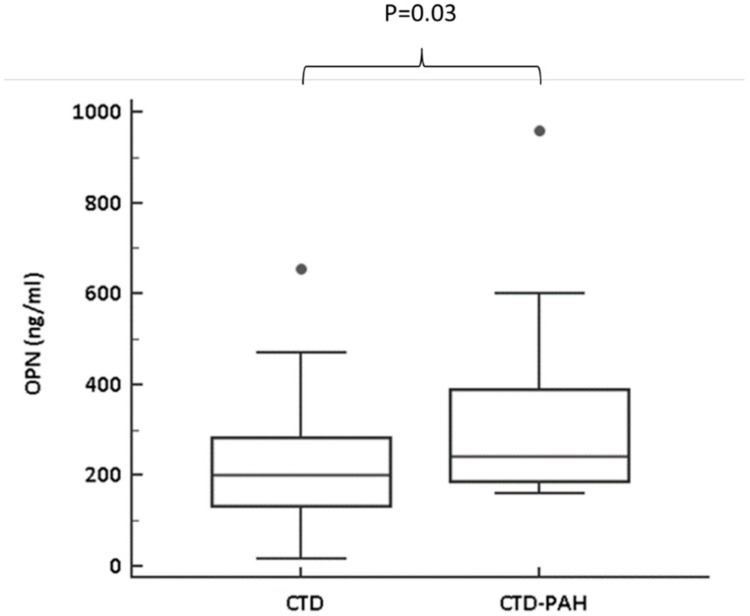
Plasma osteopontin (OPN) levels in CTD and CTD-associated pulmonary arterial hypertension (CTD-PAH) patients. As shown in the figure, CTD-PAH patients showed higher OPN plasma levels.

We then assessed the diagnostic role of OPN. First of all, patients with CTD-PAH have significantly higher plasma values compared to patients with CTD (241.0 (188.8–387.2) vs. 200.7 (133.5–281.6) ng/mL; *p* = 0.03; see also Figure 1).

**Figure 2:**
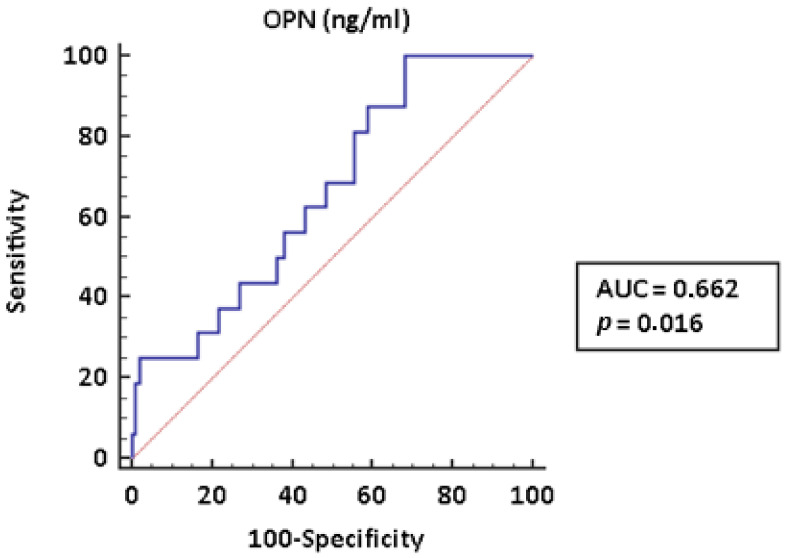
OPN ROC curve. Abbreviations: ROC—receiver operating characteristic; OPN—osteopontin; AUC—area under the curve.

To assess the diagnostic power of measuring the OPN concentration in identifying PAH in patients with CTD, we built the corresponding ROC curve. As shown in Figure 2, OPN plasma levels had an area under the curve (AUC) = 0.662 (IC95% (0.567–0.748); *p* = 0.016).

In Table 3, we report the sensitivity/specificity table for OPN.

The OPN values had a direct relationship with age (ρ = 0.249, *p* = 0.008), while were inversely related to glomerular filtration rate (ρ = −0.241, *p* = 0.01). There was no association with gender or mPAP. In a multiple regression model that had PAH as the dependent variable, and OPN, age and glomerular filtration rate as independent variables, OPN was confirmed to be independently associated with PAH diagnosis (F-ratio = 11.32, *p* < 0.0001; Table 4).

## 3. Discussion

The present data, though admittedly preliminary, show that plasma OPN levels are significantly higher in CTD-PAH than in CTD patients without PAH, independently of age and renal function. Thus, they confirm that plasma OPN is a putative biomarker of PAH worth further study in this setting. The merits and limitations of this study will be discussed in light of the current literature on the topic.

In our series, we observed a prevalence of PAH (14.2%) slightly higher than expected based on other epidemiological data (8–12%) [17]. Compared to those with CTD alone, those with a PAH diagnosis were, as expected, older and had higher PAPS. Similarly, DLCO was significantly reduced. DLCO is a well-known predictor of PAH; its alterations may antedate the recognition of pulmonary hypertension of several years [18].

To the best of our knowledge, the present is the first report to suggest a potential role for OPN in this clinical context and may also be taken as a clue for a potential pathogenetic role played by this molecule in PAH. In fact, we started from what we believe is a solid rationale. First of all, OPN was related to fibrogenesis in SSc. Patients with SSc show higher OPN plasma levels; when dermal fibroblasts are challenged with pro-fibrotic stimuli, the expression of OPN is induced, suggesting a potential role in this pathogenetic process [19]. Moreover, OPN-deficient (OPN(-/-)) mice develop less dermal fibrosis compared with wild-type (WT) mice in the bleomycin-induced dermal fibrosis model, a commonly used animal model of SSc. In vitro, OPN(-/-) dermal fibroblasts have decreased migratory capacity, and TGF-β production by OPN-deficient macrophages is reduced compared with WT animals [14]. Finally, two single nucleotide polymorphisms of the OPN gene (namely, the alleles −156G in the proximal promoter and +1239C in the untranslated region) are more frequent among SSc patients suggesting that these OPN genetic variations may contribute to SSc susceptibility [20].

Second, OPN appears potentially implicated in developing pulmonary hypertension. OPN plasma concentrations increase in patients affected by idiopathic PAH concerning healthy controls, being an independent predictor of mortality [21,22]. Similarly, OPN levels are increased in the case of pulmonary hypertension related to chronic thromboembolism, supporting the idea that this biomarker is related to developing pulmonary hypertension rather than to a specific etiology [23]. Indeed, OPN is upregulated in explanted lungs of pulmonary hypertension patients, either affected by type I or type II pulmonary hypertension, being correlated to disease severity [16]. OPN seems to be involved in the pathogenesis of the vascular remodeling process accompanying developing PAH. Fibroblasts isolated from pulmonary arteries of chronically hypoxic hypertensive calves are constitutively activated, showing a high proliferative and migratory potential. These fibroblasts overexpress OPN and its receptors. This is associated with high proliferative, migratory, and invasive properties; OPN silencing is conversely paralleled by a decreased proliferation, migration and invasion [24].

OPN levels are directly related to age and inversely related to glomerular filtration rate. This is particularly important in CTD-PAH; indeed, a lower glomerular filtration rate is observed in PAH patients, as also reported in the present study. The reduction in glomerular filtration rate is partly explained by the difference in age between the two groups; however, the altered renal function could also be the consequence of altered hemodynamics due to an overloaded right ventricle, leading to progressive functional deterioration. In any case, the association between OPN and PAH remains even after correction for age and renal function, suggesting that OPN is an independent biomarker of PAH. It should be, however, acknowledged that the association between PAH and OPN has a weak statistical significance; therefore, the differences between groups may be explained by the different glomerular filtration rates, the association, of which is much stronger. Nevertheless, our study represents a proof of concept. OPN may be proposed as a diagnostic and prognostic biomarker in CTD-PAH, the clinical relevance, of which should be assessed in further, larger cohorts. Indeed, the dosage of this protein, combined with data such as red cell distribution width (RDW), diffusing capacity of the lung for carbon monoxide (DLCO), systolic pulmonary arterial pressures (sPAP), may allow better risk stratification in CTD patients. The demonstration of the prognostic role of OPN in the context of CTD-PAH goes, however, beyond the aim of the present study and should be specifically addressed by ad hoc prospective studies.

Our study has limitations. First of all, the study population is relatively small; however, it should be acknowledged that SSc is a rare and relatively small proportion of patients who develops PAH. The AUC of the ROC curve is poor, particularly if we consider OPN in a screening strategy, requiring high sensitivity; however, our study only adds a proof of concept, which requires confirmation on a larger scale and which has possibly been underpowered by the small sample size.

A further limitation is that many patients were already receiving treatment either for PAH or for CTD-ILD, and this may have partly influenced the OPN plasma values. Moreover, not all the patients included in the present cohort underwent RHC, which was limited to those with echocardiographic findings suggestive for PAH. Ideally, being RHC the gold standard for PAH diagnosis, we cannot exclude a misclassification for a minor proportion of subjects. However, this approach is supported by international guidelines for diagnosing CTDs-PAH and is used to limit unnecessary RHC, which may expose patients to potential risks.

Finally, for the present study, the diagnosis of PAH relied on the 2015 ESC/ESR guidelines, which were revised during the 2018 PH World Symposium. The new proposed cutoff for PAH diagnosis is an mPAP ≥ 20 mmHg with a pulmonary vascular resistance ≥ 3 WU at RHC. However, it should be considered that the threshold of 25 mmHg was the diagnostic cutoff when the present study was conducted.

## 4. Materials and Methods

We performed a cross-sectional, observational study on patients already diagnosed with CTDs, referred and consecutively evaluated at the Pulmonary Hypertension Clinic of the Cardiology Division, University Hospital of Novara, from 3 October 2016 to 12 December 2019. The study protocol (no. 108/16) was approved by the local ethical committee on 9 September 2016 and conducted in strict accordance with the principles of the Declaration of Helsinki. Written informed consent was obtained from all individual participants included in the study.

The inclusion criteria were: (1) diagnosis of SSc or other connective tissue diseases at risk for PAH (MCTD, scleroderma overlap syndromes, UCTD) defined in relation to the fulfillment of diagnostic criteria international employees currently employed; (2) Age > 18.

The exclusion criteria were: (1) refusal to give informed consent to participation; (2) impossibility to undergo the investigations included in the study Protocol.

The diagnosis of SSc has been confirmed in relation to the fulfillment of the 2013 ACR/Eular classification criteria [25], while the diagnosis of MCTD was made based on Kasukawa’s criteria [26]. Patients fulfilling the classification criteria for SSc along with those of other rheumatic conditions were classified as overlap syndrome [27]. Finally, the diagnosis of UCTD was made when patients with a connective tissue disease did not meet the classification criteria of any specific syndrome [28]. We identified 113 patients who underwent:

Clinical evaluation, including a comprehensive medical history and a physical examination performed by an experienced clinician;

A biochemistry panel;

12-lead electrocardiogram with 6-limb and 6 precordial leads with paper speed set at the standard rate of 25 mm/s;

Posteroanterior and lateral chest X-rays;

Pulmonary function tests (PFTs): were performed using standardized equipment and technique with a spirometer (COSMED, Rome, Italy). The device was connected to a computer employing the software “Medisoft Expair 1.28.20”. The following standardized measurements were evaluated: forced vital capacity (FVC), forced expiratory volume in one second (FEV1), and FEV1/FVC% (also known as the Tiffeneau index). We also evaluated the diffusing capacity of the lung for carbon monoxide (DLCO), measured with the single-breath Jones–Meade protocol.

Transthoracic echocardiography (TTE) was performed using the Vivid 7 or E9 cardiovascular ultrasound machine by GE Medical Systems (Horten, Norway) with a 1.7/3.4 MHz tissue harmonic transducer. All data were obtained in standardized patient positions, according to the standards of the American Society of Echocardiography. The test was performed by an expert echocardiographer with a special interest in pulmonary hypertension. The following parameters were generated: systolic pulmonary pressure (sPAP), right atrium area (RAA), right ventricle diameter (RVD), and ejection fraction (EF). Right ventricle systolic function was evaluated by estimating the tricuspid annular plane systolic excursion (TAPSE).

According to the application of international guidelines, those patients with a suspected PAH underwent right heart catheterization within one month after TTE. PAH was defined by mean pulmonary artery pressure (mPAP) ≥ 25 mmHg, pulmonary capillary wedge pressure ≤ 15 mmHg, and pulmonary vascular resistance > 3 wood units. Whenever contraindications to RHC occurred, pulmonary hypertension was diagnosed based on echocardiography-estimated sPAP ≥ 35 mmHg and additional high probability criteria, in agreement with the 2015 ESC/ESR guidelines [1].

For each patient, a blood sample was drawn and collected in a tube with EDTA; the samples were then centrifuged at room temperature for 10 min at 3000 rpm within one hour of collection, then stored at −80 °C at the Laboratory of the University of Eastern Piedmont, Department of Medicine Translational.

OPN concentrations were measured by a commercially available enzyme-linked immunosorbent assay (ELISA) (DUOSET^®^ ELISA R&D System, Minneapolis, MN, USA. Code DY1433) following the manufacturer’s instructions.

### Statistical Analysis

Anthropometric, clinical, and biochemical data were recorded in a database and analyzed by the statistical software package MedCalc v.19.6.4 (MedCalc software, Broekstraat 52, 9030, Mariakerke, Belgium). The normality of OPN distribution was assessed by the Shapiro–Wilk test. Continuous variables are presented as medians and interquartile range (IQR). Differences in these variables between CTD and CTD-PAH patients were compared by the Mann–Whitney. Correlations between continuous variables were analyzed by Spearman’s rank test. To test the diagnostic performance of OPN in identifying patients with PAH receiver operating characteristics, curves were built with the calculation of the areas under the curve (AUC). To test whether OPN was independently associated with the diagnosis of PAH we first, run a univariate analysis evaluating the association with potential confounders, such as age, gender and renal function. We then built a multiple regression model. The level of significance chosen for all statistical analyses was 0.05 (two-tailed).

## 5. Conclusions

In conclusion, OPN was significantly associated with PAH in patients affected by SSC, suggesting a possible role for this protein as a non-invasive disease biomarker.

## Figures and Tables

**Table 1:** Clinical and laboratory features of the study population. Abbreviations: CTD—connective tissue diseases; PAH—pulmonary arterial hypertension.

Clinical Features	Study Population	CTD without PAH	CTD-PAH	*p*
Female gender	101 (89.4)	87 (89.7)	14 (87.5)	0.68
Median age, years	65.0 (54.0–75.0)	62.0 (51.0–71.0)	74.0 (69.0–78.5)	0.0004
Hydroxychloroquine	65 (57.7)	57 (58.8)	8 (50.0)	0.59
Methotrexate	15 (13.3)	15 (15.5)	0 (0.0)	0.12
Steroids	38 (33.6)	36 (37.1)	2 (12.5)	0.08
Phosphodiesterase 5 inhibitors	6 (5.3)	2 (2.1)	6 (37.5)	<0.0001
Endothelin-1 receptors antagonists	8 (7.1)	7 (7.2)	8 (50.0)	<0.0001
Riociguat	1 (0.9)	0 (0.0)	1 (6.2)	0.14
Raynaud’s phenomenon	102 (90.3)	87 (89.7)	14 (87.5)	0.61
Previous acral ulcers	49 (43.4)	43 (44.3)	7 (43.7)	1.00
Digital ulcers in the past month	5 (4.4)	4 (4.1)	1 (6.2)	0.54
Sclerodactyly	66 (58.4)	57 (58.8)	9 (56.2)	1.00
Puffy fingers	16 (14.2)	16 (16.5)	0 (0.0)	0.12
Telangiectasia	32 (28.3)	28 (28.9)	5 (31.2)	1.00
Pulmonary interstitial disease	38 (33.6)	31 (32.0)	7 (43.7)	0.40
Gastrointestinal involvement	22 (19.5)	18 (18.6)	4 (25.0)	0.51
Renal involvement	3 (2.7)	2 (2.1)	1 (6.2)	0.37
Anti-nuclear antibodies (ANA)	104 (92.0)	88 (90.7)	16 (100.0)	0.35
Anti-centromere antibodies	69 (61.1)	56 (57.7)	13 (81.2)	0.10
Anti-Scl-70 antibodies	28 (24.8)	23 (23.7)	5 (31.2)	0.54
Anti-U1-RNP antibodies	21 (18.6)	16 (16.5)	5 (31.2)	0.17
Disease duration	5 (3–13)	5 (4–13)	5 (3–11)	0.66

**Table 2:** Laboratory and instrumental data in the entire study population and in two subgroups categorized according to the presence/absence of pulmonary arterial hypertension. Abbreviations: CTD—connective tissue diseases; PAH—pulmonary arterial hypertension; WBC—white blood cells; Hb—hemoglobin—PLTs—platelets; ALT—alanine aminotransferase; AST—aspartate aminotransferase; eGFR—estimated glomerular filtration rate; CRP—C-reactive protein; ESR—erythrocyte sedimentation rate; BNP—brain natriuretic peptide; FEV1—forced expiratory volume in 1 s; FVC—forced vital capacity; TLC—total lung capacity; EF—ejection fraction; PAPS—pulmonary artery pressures; TAPSE—tricuspid annular plane excursion.

Variable	Study Population	CTD without PAH	CTD-PAH	*p*
WBC, ×10^9^/L	6.49 (5.26–7.68)	6.47 (5.26–7.63)	6.77 (5.15–7.87)	0.81
Hb, g/dL	12.8 (11.9–13.7)	12.9 (12.2–13.7)	11.3 (10.8–13.5)	0.01
PLTs, ×10^9^/L	228 (192–286)	234 (202–286)	188 (167–273)	0.07
ALT, U/L	17 (13–22)	18 (13–22)	13 (12–20)	0.33
AST, U/L	23 (20–26)	23 (20–26)	23 (20–27)	0.63
Creatinine, mg/dL	0.72 (0.61–0.88)	0.68 (0.6–0.81)	0.95 (0.81–1.08)	<0.0001
eGFR, mL/min	90 (63.5–101.3)	93 (72–103)	58.5 (51.5–64.5)	<0.0001
CRP, mg/dL	0.18 (0.04–0.78)	0.14 (0.04–0.34)	0.77 (0.04–0.98)	0.26
ESR, mm/h	14.5 (7–28)	13 (7–25)	25 (7–50)	0.31
C3, mg/dL	104 (90–121)	106 (91–121)	92 (84–119)	0.13
C4, mg/dL	24 (19–28)	24 (20–29)	22 (16–26)	0.11
BNP, pg/mL	46.8 (27.1–99.6)	39.6 (24.9–85.7)	177.0 (82.3–305.2)	<0.0001
FEV1, %	99 (88–114)	100.5 (88–113.5)	94 (88.5–113)	0.76
FVC, %	100 (90–112)	100 (90.5–113.5)	91 (80–104)	0.25
FEV1/FVC, %	109 (102–113.3)	109 (102–114)	107.5 (101–113)	0.74
TLC, %	98 (86.5–112)	99 (87–112)	82 (58–95)	0.07
DLCO-VA, %	86 (76–99)	87 (78–99)	54.5 (53–76)	0.008
DLCO-Hb, %	77 (61–91)	80 (62–91)	51 (43–72)	0.03
EF, %	63 (58–67)	63 (58–67)	61 (58.3–66)	0.41
PAPS, mmHg	27 (23–35)	26 (23–30)	44 (42–51)	<0.0001
TAPSE, mm	22 (19–24)	22 (20–24)	22 (18–23)	0.42

**Table 3:** Sensitivity/specificity table. Abbreviations: Sens—sensitivity; Spec—specificity; LR—likelihood ratio.

Criterion	Sens	95% CI	Spec	95% CI	+LR	95% CI	−LR	95% CI
>159.65	100.00	79.4–100.0	31.96	22.9–42.2	1.47	1.3–1.7	0.00	
>159.89	93.75	69.8–99.8	31.96	22.9–42.2	1.38	1.1–1.7	0.20	0.03–1.3
>174.01	87.50	61.7–98.4	41.24	31.3–51.7	1.49	1.2–1.9	0.30	0.08–1.1
>185.73	81.25	54.4–96.0	44.33	34.2–54.8	1.46	1.1–2.0	0.42	0.1–1.2
>188.15	75.00	47.6–92.7	44.33	34.2–54.8	1.35	1.0–1.9	0.56	0.2–1.4
>201.59	68.75	41.3–89.0	51.55	41.2–61.8	1.42	1.0–2.1	0.61	0.3–1.3
>225.16	62.50	35.4–84.8	56.70	46.3–66.7	1.44	0.9–2.2	0.66	0.3–1.3
>236.49	56.25	29.9–80.2	61.86	51.4–71.5	1.47	0.9–2.4	0.71	0.4–1.3
>243.8	50.00	24.7–75.3	63.92	53.5–73.4	1.39	0.8–2.4	0.78	0.5–1.3
>271.22	43.75	19.8–70.1	73.20	63.2–81.7	1.63	0.9–3.1	0.77	0.5–1.2
>293.54	37.50	15.2–64.6	78.35	68.8–86.1	1.73	0.8–3.6	0.80	0.5–1.2
>316	31.25	11.0–58.7	83.51	74.6–90.3	1.89	0.8–4.4	0.82	0.6–1.2
>450.44	25.00	7.3–52.4	97.94	92.7–99.7	12.12	2.4–60.8	0.77	0.6–1.0
>470.97	18.75	4.0–45.6	98.97	94.4–100.0	18.19	2.0–164.2	0.82	0.6–1.0
>564.24	12.50	1.6–38.3	98.97	94.4–100.0	12.13	1.2–126.1	0.88	0.7–1.1
>654.78	6.25	0.2–30.2	100.00	96.3–100.0			0.94	0.8–1.1

**Table 4:** Variables associated with PAH. In the table, we show a multiple regression model of predictive factors for PAH. Abbreviations: OPN—osteopontin; eGFR—estimated glomerular filtration rate.

Variable	Coefficient	Standard Error	r	t	*p*
OPN	0.0005	0.0002	0.19	2.09	0.04
eGFR	−0.007	0.002	−0.32	−3.52	0.0006
Age	−0.002	0.003	−0.04	−0.49	0.62

## Data Availability

All the data are available upon reasonable request to the corresponding author.
